# Measuring the Uncertainty in the Original and Negation of Evidence Using Belief Entropy for Conflict Data Fusion

**DOI:** 10.3390/e23040402

**Published:** 2021-03-28

**Authors:** Yutong Chen, Yongchuan Tang

**Affiliations:** 1 School of Computer and Information Science, Southwest University, Chongqing 400715, China; zxx200822@email.swu.edu.cn; 2 School of Big Data and Software Engineering, Chongqing University, Chongqing 401331, China

**Keywords:** Dempster-Shafer evidence theory, uncertainty management, Deng entropy, negation of basic probability assignment, data fusion

## Abstract

Dempster-Shafer (DS) evidence theory is widely used in various fields of uncertain information processing, but it may produce counterintuitive results when dealing with conflicting data. Therefore, this paper proposes a new data fusion method which combines the Deng entropy and the negation of basic probability assignment (BPA). In this method, the uncertain degree in the original BPA and the negation of BPA are considered simultaneously. The degree of uncertainty of BPA and negation of BPA is measured by the Deng entropy, and the two uncertain measurement results are integrated as the final uncertainty degree of the evidence. This new method can not only deal with the data fusion of conflicting evidence, but it can also obtain more uncertain information through the negation of BPA, which is of great help to improve the accuracy of information processing and to reduce the loss of information. We apply it to numerical examples and fault diagnosis experiments to verify the effectiveness and superiority of the method. In addition, some open issues existing in current work, such as the limitations of the Dempster-Shafer theory (DST) under the open world assumption and the necessary properties of uncertainty measurement methods, are also discussed in this paper.

## 1. Introduction

Information fusion, as a fusion method that uses normalization and aggregation functions to compare large amounts of data, is the key to data fusion technology [1]. In recent years, it has been widely used in condition identification [2,3], location discovery [4] and other fields [5,6]. With the increasing complexity of systems, there are many restrictions on relying on a single sensor for monitoring. Compared with single-source independent processing, information fusion has the advantages of improving detection and reliability, reducing inference ambiguity, and improving detection accuracy and performance [7]. However, due to the uncertainty of the real world, multi-sensor information sources may be affected by the environment. Therefore, the information in practical applications is often uncertain and imprecise [8]. According to the data type with uncertainty and the type of uncertainty, three different types of uncertainty caused by fuzziness, randomness and partial information are identified [9]. Many methods have been proposed to deal with the uncertain information, such as rough sets theory [10,11], fuzzy set theory [12], Dempster-Shafer (DS) evidence theory [13,14], D number [15,16], R number [17], and so on [18,19].

The Dempster-Shafer evidence theory was first proposed by Dempster [13] in 1967, and then further developed by his student Shafer [14] in 1976. This theory is a reasoning theory that can effectively deal with uncertain information [20,21], and is widely used in many fields, such as fault diagnosis [22,23], decision making [24,25,26], risk assessment [27,28], classification [29,30,31], and so on [32,33], which solves many problems caused by uncertain information. However, in application, the classic combination rule of DS theory has been found to have some problems. For example, when it is used to fuse highly conflicting evidence [34], the result may be counterintuitive [35,36,37], which makes many researchers question the effectiveness of the theory when it is used to merge highly conflicting data [38]. Therefore, how to deal with the uncertainty under the framework of DS theory is still an open issue [39,40,41].

Shannon entropy is a well-known uncertainty measurement theory, which can effectively use probability distribution to measure uncertainty. It has been widely used in many fields, such as the contrast between trade-offs in social conflict analysis [42]. This entropy-based measurement has attracted wide attention from researchers [43,44], and provides new ideas for researchers to solve the uncertainty measurement problem under the DS evidence theory framework. The Deng entropy [45] is a generalization of Shannon entropy, which can also be regarded as a generalized Shannon entropy. Deng entropy is proposed to measure the uncertainty of basic probability assignment. When basic probability assignment (BPA) degenerates to a probability distribution, Deng entropy degenerates to Shannon entropy.

The concept of Negation of BPA is considered to be of great significance for uncertainty measurement and knowledge expression. Yin et al. [46] proposed a more general method to find the negation of BPA. Based on this, this paper proposes an improved data fusion method which combines negation of BPA and Deng entropy, and takes them together as the uncertain degree. The method first uses Deng entropy to measure the uncertainty of evidence, then calculates the negation of BPA, and then calculates the uncertainty of the negation of BPA through Deng entropy to achieve the effect of obtaining more uncertain information. After that, combine the results of the two uncertainty measurements to get the final body uncertainty, and use the uncertainty to correct the evidence. Finally, the DS theory combination rules are used to fuse the revised evidence.

The rest of this paper is organized as follows. The second section briefly introduces the Dempster-Shafer evidence theory, Shannon entropy, Deng entropy and the relevant knowledge of the BPA. The third section proposes an improved data fusion method based on Deng entropy and negation of BPA. The fourth section verifies the method through a numerical example. In the fifth section, this method is applied to an actual fault detection experiment and compared with other methods. In the sixth section, we discussed some open issues in future work. Finally, the seventh section gives a conclusion.

## 2. Preliminaries

In this section, we will briefly introduce some preliminaries. 

### 2.1. Dempster-Shafer Evidence Theory

There is a non-empty set $\Omega =\{\theta_{1},\;\theta_{2},\ldots ,\;\theta_{i},\ldots ,\;\theta_{n}\}$, which contains N mutually exclusive and exhaustive events. Such Ω is called the frame of discernment (FOD). The power set of Ω contains 2^N^ elements, expressed as follows:

(1) $$2^{\Omega }=\;\{\emptyset ,\;\left\{\theta_{1}\right\},\;\left\{\theta_{2}\right\},\;...,\;\left\{\theta_{n}\right\},\;\left\{\theta_{1},\;\theta_{2}\right\},\;...,\;\left\{\theta_{1},\;\theta_{2},\;...,\;\theta_{i}\right\},\;...,\;\Omega \}.$$

In the frame of discernment, define mass probability function *m*; it is a function from the power set 2^Ω^ to [0, 1], and the function satisfies the following relationship:

(2) $$m(\emptyset )=0,\underset{A\in \Omega }{\sum }m\left(A\right)=1.$$

When *m(A)* > 0, the proposition subset A is called a focal element. *m(A)* is the mass function value of A, also known as basic probability assignment (BPA) or basic belief assignment (BBA). 

The body of evidence (BOE) is considered to be the unit of uncertain information evidence, which consists of focal sets and their mass value, expressed as follows:

(3) $$(ℜ,m)=\{\langle A,m(A)\rangle :A\in 2^{\Omega },m(A)>0\}.$$

where, ℜ is a subset of the powerset 2^Ω^.

A mass function m can also be expressed as a belief function Bel or a plausibility function Pl, which is defined as follows:

(4) $$Bel(A)=\sum_{\emptyset \neq B\subseteq A}^{}m(B),Pl(A)=\sum_{B\cap A\neq \emptyset }^{}m(B).$$

Belief function *Bel(A)* of subset A represents the level of support for proposition A, and the plausibility function *Pl(A)* represents the degree of no objection to proposition A.

In the framework of evidence theory, two independent mass functions *m*_1_, *m*_2_ can be data fused by the following Dempster combination rules [13,14]:

(5) $$m(A)=(m_{1}⊕m_{2})(A)=\frac{1}{1-k}\sum_{B\cap C=A}^{}m_{1}(B)m_{2}(C).$$

In the formula, *k* is called the normalization factor and is defined as:

(6) $$k=\sum_{B\cap C=\emptyset }^{}m_{1}(B)m_{2}(C).$$

### 2.2. Shannon Entropy

In 1948, Claude Shannon first proposed the concept of “Information Entropy” and began to quantify information. Shannon entropy shows how much the measurement of information is equal to the uncertainty, which effectively solves the uncertainty measurement problem of probability measurement.

For the discrete probability set *p*_1_,..., *p_n_*, Shannon entropy is defined as follows [47]:

(7) $$H=-\sum_{}^{}p_{i}\log p_{i}.$$

### 2.3. Belief Entropy

As a belief entropy, Deng entropy is a promotion of Shannon entropy. It is similar in form to the classical Shannon entropy, but it deals with the belief for each focal element, and provides some effective method for uncertainty measurement and processing more uncertainty information.

Deng entropy is defined as follow [45]:

(8) $$E_{d}(m)=-\sum_{A\subseteq X}^{}m(A)\log_{2}\frac{m(A)}{2^{\left|A\right|}-1}.$$

where *m* is a mass function, *A* is the focal element of *m*, and |*A*| is the cardinality of A.

After a simple transformation, the Deng entropy can be regarded as a composite measure:

(9) $$E_{d}(m)=\sum_{A\subseteq X}^{}m(A)\log_{2}(2^{\left|A\right|}-1)-\sum_{A\subseteq X}^{}m(A)\log_{2}m(A).$$

### 2.4. The Negation of BPA

Expressing information in a negative way is important in the field of information science. After Zadeh formally proposed the negation of probabilistic events in the BISC blog, Smets [48] used the implacability function and commonality function to define the negation of the mass function, and used $\bar{m}$ to represent the negation of m in the model, expressed as $\bar{m}(A)=m(\bar{A})$.

However, the Smets model has limitations. When applied to the negation of m(θ), $m(\bar{\theta })$ is always equal to 0. At the same time, the Dempster-Shafer theory (DST) has a more general framework than the Bayes structure, and the BPA is easier to obtain. Therefore, Yin et al. [46] proposed a method to calculate the negation of the basic probability assignment. This method takes into account the number of focal elements, and the negations of focal elements are independent of each other. 

In the frame of discernment, for each focal element *e_i_*, replace the initial belief assignment *p_i_* with complementary probability 1 − *p_i_* to obtain the negation of *m*(*e_i_*). Next, calculate the sum σ of $\bar{m}(e_{i})$ of all focal elements, and then perform normalization.

After the above series of transformations, the general formula of negation of the mass function is derived as:

(10) $$\bar{m}(e_{i})=\frac{1-m(e_{i})}{n-1}.$$

where *n* is the number of focal elements.

## 3. The Proposed Data Fusion Method

Aiming at the problem of uncertainty in the FOD, in this section, this paper proposes an improved data fusion method based on the Deng entropy and the negation of BPA.

In this method, the uncertainty of BPA and the negation of BPA are measured by the Deng entropy, and the BPA in BOE is modified by uncertainty; then the data fusion is performed by the DS combination rule. Finally, the fusion results are used as the basis for decision-making.

**Figure 1:**
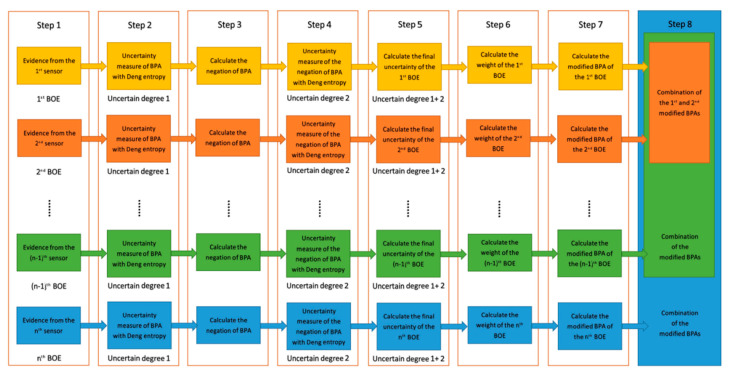
An improved data fusion method using Deng entropy to measure the uncertainty in basic probability assignment (BPA) and the negation of BPA.

The data fusion process of this method is shown in Figure 1.

The details of the data fusion steps are as follows:

*Step 1:* Data obtained from sensors is modeled as BOE.

Due to the uncertainty of the real world, the knowledge contained in information sources is often difficult to extract. Considering that BPA can be applied to practical problems, the data from different sensors will be modeled as body of evidence (BOE), and their BPA values will be obtained.

*Step 2:* Uncertainty measure of BPA with Deng entropy.

After obtaining the data from sensors, it is necessary to measure the uncertainty of the original data. In this paper, by calculating the Deng entropy of the BPA value of each BOE, the uncertainty of the initial evidence is obtained, which is uncertain degree 1.

For the *i*-th BOE (*i* = 1, 2, …, *m*), the uncertainty of the BPA value is calculated as follows:

(11) $$E_{d}(m_{i})=-\sum_{A\subseteq X}^{}m_{i}(A)\log_{2}\frac{m_{i}(A)}{2^{\left|A\right|}-1}.$$

where *m* is a mass function, *A* is the focal element of *m*, and |*A*| is the cardinality of *A*.

*Step 3:* Calculate the negation of BPA.

In order to obtain more uncertain information, this paper uses Yin et al.’s method to negate the information. In each obtained BOE, for the mass function *m*(*e_i_*) of the *i*-th focal element *e_i_*, negate according to the following formula to obtain the negation of BPA:

(12) $$\bar{m}\left(e_{i}\right)=\frac{1-m\left(e_{i}\right)}{n-1}.$$

where *n* is the number of focal elements.

*Step 4:* Uncertainty measure of the negation of BPA with Deng entropy.

Combining the negation of BPA and Deng entropy, the new uncertain degree is calculated. This step will measure the uncertainty of the inverse evidence to realize the combination of the two. For the negation of BPA obtained in the third step, continue to use the Deng entropy to measure the degree of uncertainty, and record the result as uncertainty degree 2.

For the focal elements’ negation of BPA $\bar{m}$ of the *i*-th BOE (*i* = 1, 2, …, *m*), the uncertainty corresponding to the Deng entropy is calculated as follows:

(13) $$E_{d}(m_{i})=-\sum_{A\subseteq X}^{}{\bar{m}}_{i}(A)\log_{2}\frac{{\bar{m}}_{i}(A)}{2^{\left|A\right|}-1}.$$

*Step 5:* Calculate the final uncertainty of BOE.

Before further processing of the data, the final BOE uncertainty needs to be calculated. Considering the uncertainty degree of the original BPA and the negation of BPA, the final uncertainty will be more accurate.

Suppose $E_{du}\left(m_{i}\right)$ represents the final degree of uncertainty of the *i*-th group of data. In this step, this paper adds up the data uncertainty measured in the second and fourth steps to obtain the new evidence uncertainty measurement results $E_{du}\left(m_{i}\right)$.

*Step 6:* Calculate the weight of each BOE.

Based on the new degree of uncertainty calculated in the fifth step, for the *i*-th BOE (*i* = 1, 2, …, *m*), the weight *w_i_* is calculated by the following formula:

(14) $$w_{i}=\frac{E_{du}\left(m_{i}\right)}{\sum_{i=1}^{m}E_{du}\left(m_{i}\right)}.$$

*Step 7:* Calculate the modified BPA.

Before final data fusion, modify the BPA value in BOE with BOE uncertain degree to obtain modified BPA.

For Proposition A in each BOE, the modified BPA is calculated as follows:

(15) $$m_{w}\left(A\right)=\overset{m}{\underset{i=1}{\sum }}w_{i}m_{i}\left(A\right).$$

*Step 8:* Use Dempster’s rule to combine modified BPAs.

Finally, use the classic combination rule of DS theory to fuse the modified BPA obtained in the seventh step.

For each proposition A in BOE, the fusion result can be obtained by calculating the Dempster combination rule (*m* − 1) times by the following formula:

(16) $$m\left(A\right)={\left({\left({\left({\left(m_{w}⊕m_{w}\right)}_{1}⊕m_{w}\right)}_{2}\ldots ⊕m_{w}\right)}_{\left(m-2\right)}⊕m_{w}\right)}_{\left(m-1\right)}(A),\;m\geq 2.$$

## 4. Numerical Example

In order to verify the effectiveness of the data fusion method proposed in this paper and to facilitate comparison with other methods, this part reviews the experiments in [49], and realizes the verification and comparison of the method in this paper through a numerical example.

In this example, the evidence reported by five sensors is modeled as BPA, which is shown in Table 1 as m_1_, m_2_, m_3_, m_4_, and m_5_. Intuitively, target A may be the right target with the highest credibility.

For this example, perform the method in Section 3.

In the 1st step, get the data from the sensor and model it as BOE. In this example, the value in [49] is used, which is shown in Table 1.

In the 2nd step, according to Deng entropy’s Formula (11), the uncertainty of BPA of each sensor is calculated as follows:

$$E_{d}\left(m_{1}\right)=-\sum_{A\subseteq X}^{}m_{1}\left(A\right)\log_{2}\frac{m_{1}\left(A\right)}{2^{\left|A\right|}-1}=1.5664$$

$$E_{d}\left(m_{2}\right)=-\sum_{A\subseteq X}^{}m_{2}\left(A\right)\log_{2}\frac{m_{2}\left(A\right)}{2^{\left|A\right|}-1}=0.4690$$

$$E_{d}\left(m_{3}\right)=-\sum_{A\subseteq X}^{}m_{3}\left(A\right)\log_{2}\frac{m_{3}\left(A\right)}{2^{\left|A\right|}-1}=1.8092$$

$$E_{d}\left(m_{4}\right)=-\sum_{A\subseteq X}^{}m_{4}\left(A\right)\log_{2}\frac{m_{4}\left(A\right)}{2^{\left|A\right|}-1}=1.8914$$

$$E_{d}\left(m_{5}\right)=-\sum_{A\subseteq X}^{}m_{5}\left(A\right)\log_{2}\frac{m_{5}\left(A\right)}{2^{\left|A\right|}-1}=1.7710$$

In the 3rd step, calculate the negation of BPA from the first sensor according to the Formula (12):

$$\bar{m}\left(A\right)=\frac{1-m\left(A\right)}{n-1}=0.295$$

$$\bar{m}\left(B\right)=\frac{1-m\left(B\right)}{n-1}=0.355$$

$$\bar{m}\left(C\right)=\frac{1-m\left(C\right)}{n-1}=0.350$$

Similarly, Formula (12) is used to calculate the negation of BPA of the 2nd to 5th sensors, and the results are shown in Table 2:

In the 4th step, use Formula (13) to calculate the Deng entry of the negation of BPA, and obtain the value of uncertain degree 2 as follows:

$$E_{d}\left({\bar{m}}_{1}\right)=-\underset{A\subseteq x}{\sum }{\bar{m}}_{1}\left(A\right)\log_{2}\frac{{\bar{m}}_{1}\left(A\right)}{2^{\left|A\right|}-1}=1.5801$$

$$E_{d}\left({\bar{m}}_{2}\right)=-\underset{A\subseteq x}{\sum }{\bar{m}}_{2}\left(A\right)\log_{2}\frac{{\bar{m}}_{2}\left(A\right)}{2^{\left|A\right|}-1}=0.4690$$

$$E_{d}\left({\bar{m}}_{3}\right)=-\underset{A\subseteq x}{\sum }{\bar{m}}_{3}\left(A\right)\log_{2}\frac{{\bar{m}}_{3}\left(A\right)}{2^{\left|A\right|}-1}=2.0286$$

$$E_{d}\left({\bar{m}}_{4}\right)=-\underset{A\subseteq x}{\sum }{\bar{m}}_{4}\left(A\right)\log_{2}\frac{{\bar{m}}_{4}\left(A\right)}{2^{\left|A\right|}-1}=2.0447$$

In the 5th step, the uncertainty degree of BOE calculated in the 2nd step and the 4th step is added to obtain the final uncertainty degree of BOE as follows:

$$E_{du}\left(m_{1}\right)=1.5664+1.5801=3.1465$$

$$E_{du}\left(m_{2}\right)=0.4690+0.4690=0.9380$$

$$E_{du}\left(m_{3}\right)=1.8092+2.0286=3.8378$$

$$E_{du}\left(m_{4}\right)=1.8914+2.0447=3.9361$$

$$E_{du}\left(m_{5}\right)=1.7710+2.0676=3.8386$$

In the 6th step, the weight of each BOE is calculated by Formula (14). The results are as follows:

$$w_{1}=\frac{E_{du}\left(m_{1}\right)}{\sum_{i=1}^{5}E_{du}\left(m_{1}\right)}=0.2005$$

$$w_{2}=\frac{E_{du}\left(m_{2}\right)}{\sum_{i=1}^{5}E_{du}\left(m_{2}\right)}=0.0598$$

$$w_{3}=\frac{E_{du}\left(m_{3}\right)}{\sum_{i=1}^{5}E_{du}\left(m_{3}\right)}=0.2445$$

$$w_{4}=\frac{E_{du}\left(m_{4}\right)}{\sum_{i=1}^{5}E_{du}\left(m_{4}\right)}=0.2508$$

$$w_{5}=\frac{E_{du}\left(m_{5}\right)}{\sum_{i=1}^{5}E_{du}\left(m_{5}\right)}=0.2445$$

In the 7th step, with Equation (15), the modified BPA is calculated as follows:

$$m_{w}\left(A\right)=\overset{5}{\underset{i=1}{\sum }}w_{i}m_{i}\left(A\right)=0.5087$$

$$m_{w}\left(B\right)=\overset{5}{\underset{i=1}{\sum }}w_{i}m_{i}\left(B\right)=0.1786$$

$$m_{w}\left(C\right)=\overset{5}{\underset{i=1}{\sum }}w_{i}m_{i}\left(C\right)=0.0661$$

$$m_{w}\left(A,C\right)=\overset{5}{\underset{i=1}{\sum }}w_{i}m_{i}\left(A,C\right)=0.2467$$

In the 8th step, data fusion is carried out based on the Dempster combination rules (16):

$$m\left(A\right)={\left({\left({\left({\left(m_{w}⊕m_{w}\right)}_{1}⊕m_{w}\right)}_{2}⊕m_{w}\right)}_{3}⊕m_{w}\right)}_{4}\left(A\right)=0.9887$$

$$m\left(B\right)={\left({\left({\left({\left(m_{w}⊕m_{w}\right)}_{1}⊕m_{w}\right)}_{2}⊕m_{w}\right)}_{3}⊕m_{w}\right)}_{4}\left(B\right)=0.0007$$

$$m\left(C\right)={\left({\left({\left({\left(m_{w}⊕m_{w}\right)}_{1}⊕m_{w}\right)}_{2}⊕m_{w}\right)}_{3}⊕m_{w}\right)}_{4}\left(C\right)=0.0084$$

$$m\left(A,C\right)={\left({\left({\left({\left(m_{w}⊕m_{w}\right)}_{1}⊕m_{w}\right)}_{2}⊕m_{w}\right)}_{3}⊕m_{w}\right)}_{4}\left(A,C\right)=0.0037$$

**Figure 2:**
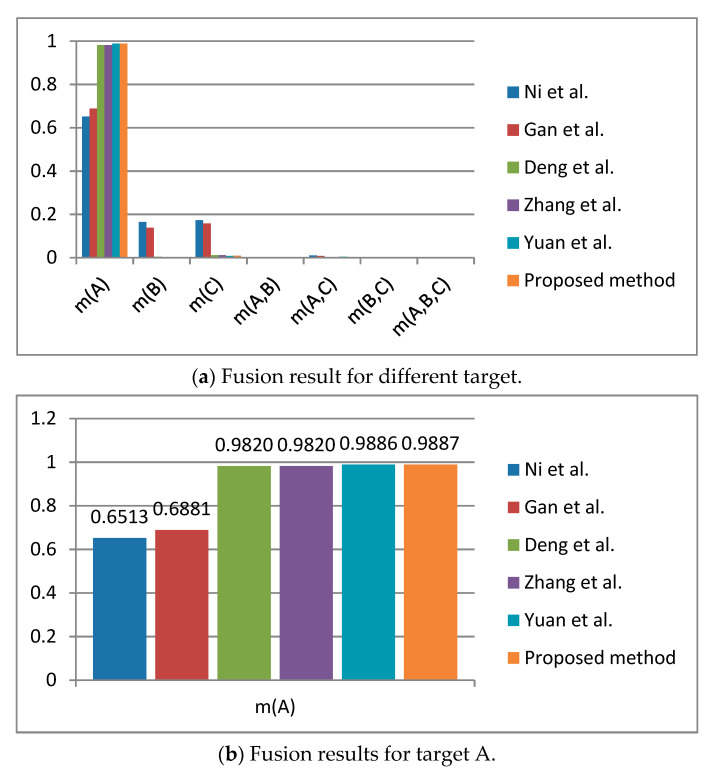
Comparison of fusion results of different methods in the numerical example.

The fusion results of other methods are shown in Table 3, and they are simply compared in the form of a bar chart in Figure 2.

In the proposed method, appropriate data preprocessing is applied before the final data fusion, such as using the Deng entropy to measure the uncertainty of information, which is conducive to the fusion of conflicting data. Therefore, according to the experimental results of this method, even if the second group of evidence has a big conflict with other evidence, we can still infer that A is the correct target, which is in line with our intuition and proves the effectiveness of this method. At the same time, in comparison with other fusion methods, we found that the methods of Ni et al.’s [50] and Gan et al.’s [51] carried out uncertainty measurement and data fusion for the subset of propositions that are not needed for decision-making. The process is more cumbersome and will lead to dispersal of confidence. The method proposed in this paper avoids this problem, makes the fusion result more credible, and facilitates decision-making. Compared with the other methods mentioned, this method takes more uncertainty into account in the process of negation of BPA, which makes the measurement results more accurate.

## 5. Application

In this section, taking the fault diagnosis experiment of a motor rotor in [54] as an example, the proposed method is applied to the engineering application program that needs decision analysis to verify the effectiveness of the method.

### 5.1. Problem Statement

According to the experimental content in [54], there are three different fault modes of the motor rotor, which are {F1} = {rotor unbalance}, {F2} = {rotor misalignment}, {F3} = {support loosening}. In this experiment, vibration acceleration sensors are placed in different positions to collect vibration signals, and the amplitude of the acceleration vibration frequency under three different frequencies of Freq1, Freq2 and Freq3 is defined as the fault characteristic variable. Table 4 shows the data from three vibration acceleration sensors m_s_1__(·), m_s_2__(·) and m_s_3__(·).

### 5.2. Data Fusion Procedure Based on the Proposed Method

According to the method mentioned in Section 3 of this paper, the data processing proceeds as follows:

*Step 1:* Data obtained from sensors is modeled as BOE.

This section directly uses the fault diagnosis data modeled as BPA in [54] and shows it in Table 4. For the detailed process of modeling sensor data as BPA in Table 4, please refer to [54].

*Step 2:* Uncertainty measure of BPA with the Deng entropy.

In this paper, we use the Deng entropy to measure uncertainty, and use Formula (11) to calculate the Deng entropy of each BOE. The detailed calculation process of the Deng entropy of the BPA value at Freq1 vibration acceleration frequency is shown as follows:

$$E_{d}\left(m_{s_{1}}\right)=-\sum_{A\subseteq X}^{}m_{s_{1}}\left(A\right)\log_{2}\frac{m_{s_{1}}\left(A\right)}{2^{\left|A\right|}-1}=1.1196$$

$$E_{d}\left(m_{s_{2}}\right)=-\sum_{A\subseteq X}^{}m_{s_{2}}\left(A\right)\log_{2}\frac{m_{s_{2}}\left(A\right)}{2^{\left|A\right|}-1}=2.3975$$

$$E_{d}\left(m_{s_{3}}\right)=-\sum_{A\subseteq X}^{}m_{s_{3}}\left(A\right)\log_{2}\frac{m_{s_{3}}\left(A\right)}{2^{\left|A\right|}-1}=3.0161$$

The Deng entropy of BOE at Freq2 and Freq3 vibration acceleration frequencies is also calculated and shown in Table 5.

*Step 3:* Calculate the negation of BPA.

In this paper, the general method of Yin et al. is used to calculate the negation of BPA. Through Formula (12), for Freq1 vibration acceleration frequency, each negation of BPA is calculated as follows:

$${\bar{m}}_{s_{1}}\left(\left\{F2\right\}\right)=\frac{1-m_{s_{1}}\left(\left\{F2\right\}\right)}{n-1}=0.0608$$

$${\bar{m}}_{s_{1}}\left(\left\{F3\right\}\right)=\frac{1-m_{s_{1}}\left(\left\{F3\right\}\right)}{n-1}=0.3332$$

$${\bar{m}}_{s_{1}}\left(\left\{F1,F2\right\}\right)=\frac{1-m_{s_{1}}\left(\left\{F1,F2\right\}\right)}{n-1}=0.2816$$

$${\bar{m}}_{s_{1}}\left(\left\{F1,F2,F3\right\}\right)=\frac{1-m_{s_{1}}\left(\left\{F1,F2,F3\right\}\right)}{n-1}=0.3244$$

$${\bar{m}}_{s_{2}}\left(\left\{F2\right\}\right)=\frac{1-m_{s_{2}}\left(\left\{F2\right\}\right)}{n-1}=0.1447$$

$${\bar{m}}_{s_{2}}\left(\left\{F3\right\}\right)=\frac{1-m_{s_{2}}\left(\left\{F3\right\}\right)}{n-1}=0.3330$$

$${\bar{m}}_{s_{2}}\left(\left\{F1,F2\right\}\right)=\frac{1-m_{s_{2}}\left(\left\{F1,F2\right\}\right)}{n-1}=0.3118$$

$${\bar{m}}_{s_{2}}\left(\left\{F1,F2,F3\right\}\right)=\frac{1-m_{s_{2}}\left(\left\{F1,F2,F3\right\}\right)}{n-1}=0.2104$$

$${\bar{m}}_{s_{3}}\left(\left\{F2\right\}\right)=\frac{1-m_{s_{3}}\left(\left\{F2\right\}\right)}{n-1}=0.2532$$

$${\bar{m}}_{s_{3}}\left(\left\{F3\right\}\right)=\frac{1-m_{s_{3}}\left(\left\{F3\right\}\right)}{n-1}=0.3332$$

$${\bar{m}}_{s_{3}}\left(\left\{F1,F2\right\}\right)=\frac{1-m_{s_{3}}\left(\left\{F1,F2\right\}\right)}{n-1}=0.3286$$

$${\bar{m}}_{s_{3}}\left(\left\{F1,F2,F3\right\}\right)=\frac{1-m_{s_{3}}\left(\left\{F1,F2,F3\right\}\right)}{n-1}=0.0849$$

Using the same method, we can calculate the negation of BPA under Freq2 and Freq3, and the results are shown in Table 6.

*Step 4:* Uncertainty measure of the negation of BPA with the Deng entropy.

In this step, we calculate the Deng entropy of the negation of BPA to obtain more uncertain information. With Equation (13), the Deng entropy of the negation BPA at Freq1 vibration acceleration frequency is calculated as follows:

$$E_{d}\left({\bar{m}}_{s_{1}}\right)=-\sum_{A\subseteq X}^{}{\bar{m}}_{s_{1}}\left(A\right)\log_{2}\frac{{\bar{m}}_{s_{1}}\left(A\right)}{2^{\left|A\right|}-1}=3.1727$$

$$E_{d}\left({\bar{m}}_{s_{2}}\right)=-\sum_{A\subseteq X}^{}{\bar{m}}_{s_{2}}\left(A\right)\log_{2}\frac{{\bar{m}}_{s_{2}}\left(A\right)}{2^{\left|A\right|}-1}=3.0141$$

$$E_{d}\left({\bar{m}}_{s_{3}}\right)=-\sum_{A\subseteq X}^{}{\bar{m}}_{s_{3}}\left(A\right)\log_{2}\frac{{\bar{m}}_{s_{3}}\left(A\right)}{2^{\left|A\right|}-1}=2.6189$$

Similarly, the Deng entropy of the negation of the BPA under Freq2 and Freq3 can be calculated. The results are shown in Table 7.

*Step 5:* Calculate the final uncertainty of BOE.

The final uncertainty of BOE is based on two measurements of BPA and the negation of BPA by the Deng entropy. In this step, the two uncertainty degrees of BOE under Freq1 are added to obtain the final uncertainty degree $E_{du}$:

$$E_{du}\left(m_{s_{1}}\right)=1.1196+3.1727=4.2923$$

$$E_{du}\left(m_{s_{2}}\right)=2.3975+3.0141=5.4116$$

$$E_{du}\left(m_{s_{3}}\right)=3.0161+2.6189=5.6350$$

The final uncertain degree of BOE under Freq2 and Freq3 is shown in Table 8.

*Step 6:* Calculate the weight of each BOE.

Through Formula (14), the weight of each BOE under the Freq1 vibration acceleration frequency is calculated as follows:

$$w_{s_{1}}=\frac{E_{du}\left(m_{s_{1}}\right)}{\sum_{i=1}^{3}E_{du}\left(m_{s_{1}}\right)}=0.2798$$

$$w_{s_{2}}=\frac{E_{du}\left(m_{s_{2}}\right)}{\sum_{i=1}^{3}E_{du}\left(m_{s_{2}}\right)}=0.3528$$

$$w_{s_{3}}=\frac{E_{du}\left(m_{s_{3}}\right)}{\sum_{i=1}^{3}E_{du}\left(m_{s_{3}}\right)}=0.3674$$

For the vibration acceleration frequencies of Freq2 and Freq3, the weight of each BOE is shown in Table 9.

*Step 7:* Calculate the modified BPA.

According to Equation (15), the modified BPA under Freq1 can be calculated as follows:

$$m_{w}\left(\left\{F2\right\}\right)=\overset{3}{\underset{i=1}{\sum }}w_{s_{i}}m_{i}\left(\left\{F2\right\}\right)=0.5167$$

$$m_{w}\left(\left\{F3\right\}\right)=\overset{3}{\underset{i=1}{\sum }}w_{s_{i}}m_{i}\left(\left\{F3\right\}\right)=0.0005$$

$$m_{w}\left(\left\{F1,F2\right\}\right)=\overset{3}{\underset{i=1}{\sum }}w_{s_{i}}m_{i}\left(\left\{F1,F2\right\}\right)=0.0714$$

$$m_{w}\left(\left\{F1,F2,F3\right\}\right)=\overset{3}{\underset{i=1}{\sum }}w_{s_{i}}m_{i}\left(\left\{F1,F2,F3\right\}\right)=0.4114$$

The modified BPA of Freq2 and Freq3 can also be calculated by Equation (15), and the results are shown in Table 10.

*Step 8:* Use Dempster’s rule to combine modified BPAs.

By using Equation (16), the modified BPA of Freq1 is fused as follows:

$$m\left(\left\{F2\right\}\right)=(({\left({\left(m_{w}⊕m_{w}\right)}_{1}⊕m_{w}\right)}_{2}\left(\left\{F2\right\}\right)=0.8871$$

$$m\left(\left\{F3\right\}\right)=(({\left({\left(m_{w}⊕m_{w}\right)}_{1}⊕m_{w}\right)}_{2}\left(\left\{F3\right\}\right)=0.0002$$

$$m\left(\left\{F1,F2\right\}\right)=(({\left({\left(m_{w}⊕m_{w}\right)}_{1}⊕m_{w}\right)}_{2}\left(\left\{F1,F2\right\}\right)=0.0430$$

$$m\left(\left\{F1,F2,F3\right\}\right)=(({\left({\left(m_{w}⊕m_{w}\right)}_{1}⊕m_{w}\right)}_{2}\left(\left\{F1,F2,F3\right\}\right)=0.0697$$

Table 11 shows the fusion results of Freq2 and Freq3.

### 5.3. Discussion

**Figure 3:**
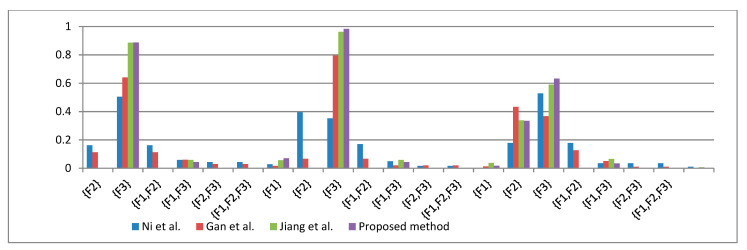
Data fusion results of different methods for each proposition of fault type.

The following tables and Figure 3 show the data fusion results under different methods.

According to Table 11, Table 12, Table 13 and Table 14, the proposed method has consistent diagnostic results with other methods, that is, F2 has the highest confidence at any test frequency. Therefore, the fault diagnosis experiment results show that the fault type is F2, and the effectiveness of the proposed method is also verified. In addition, according to Table 12, Table 13 and Table 14 and Figure 3, we can find that the method proposed in this paper has higher confidence for F2 than the data fusion method proposed in the literature [50,51,54], and it effectively avoids the confidence dispersion existing in the literature [50,51], which leads to the problem that is not conducive to decision making. This is because the proposed method obtains more uncertain information through the negation of BPA, improves the accuracy of information processing, and reduces the loss of information. In addition, this method combines the Deng entropy with the negation BPA, which also ensures the ability to deal with conflicting data.

## 6. Open Issues for Future Work

In the framework of DST, this paper proposes a new data fusion method that combines the Deng entropy and the negation of BPA, which can effectively deal with conflicts in the data fusion of evidence and improve the accuracy of information processing. However, it should be noted that this method is based on the framework of evidence theory and uses Deng entropy to measure the uncertainty of information. Although the fusion method is optimized, there are still some open issues in DST and Deng entropy.

First, the application of the classical Dempster-Shafer evidence theory has limitations. The classical DST is defined under the closed world hypothesis, under which the focal element of evidence theory does not include the empty set mass function. This type of “incomplete information” is missed by the uncertainty information classification under the closed world assumption, leading to some open issues. According to the open world hypothesis [55,56,57], many new methods of uncertainty measurement and data fusion have been proposed [58,59], but there is still no universally accepted uncertainty quantification method. In the process of further research in this area, we suggest that the open world assumption should be considered more comprehensively.

Second, open issues exist in the properties of Deng entropy. In literature [60], the five properties that must be verified by the total uncertain measure (TU) established by Klir and Wierman [61] are used to analyze the Deng entropy, and [62] also verifies the properties of some improved Deng entropy. It is found that Deng entropy only satisfies the probabilistic consistency among the five properties. Therefore, all the properties mentioned in [59,63,64] should be fully considered in future research on the improved or proposed methods of uncertainty measurement.

Finally, under the closed world and open world assumptions, a belief entropy or uncertainty measure has no universally accepted properties, and some existing properties are considered controversial. For example, for a theoretical measurement that distinguishes two uncertainties of discord and non-specificity, the range attribute is considered questionable. So far, only the upper entropy measure can satisfy the basic properties proposed, while for other measurement methods, it is difficult to verify all the attributes, which affects the development of new reliability entropy or measurement methods. Therefore, we believe that in the following work, some new properties should be obeyed by the measurement method.

## 7. Conclusions

The method of studying negation can obtain uncertainty information of evidence from a new perspective; the uncertainty in the evidence and the negation of evidence should be addressed simultaneously. Therefore, this paper proposes an improved data fusion method based on Deng entropy for measuring the uncertain degree in BPA and the negation of BPA. 

This method constructs a new uncertainty measurement strategy, which takes the original BPA’s Deng entropy as the first part of the uncertainty degree and the negation of BPA’s Deng entropy as the second part of the uncertainty degree, and finally aggregates them together as the final uncertainty degree of the BOE. This strategy enables the method to overcome the data conflicts reported by sensors, and at the same time to consider more uncertainties. It has the advantages of improving the accuracy of information processing and reducing the loss of information. On this basis, this method modifies the evidence according to the uncertainty measurement results, and uses DS classical combination rules for data fusion. While using weights to modify the evidence, this method retains the combination rule framework of Dempster-Shafer theory, and has the advantages of generality and additivity of the Dempster-Shafer theory. Finally, the method is applied to numerical examples and fault detection experiments, and the experimental results verify the effectiveness and superiority of the method.

## Figures and Tables

**Table 1:** BPAs in the numerical example.

BPA	m(A)	m(B)	m(C)	m(A,C)
1st Sensor report: m1 (·)	0.41	0.29	0.3	0
2nd Sensor report: m2 (·)	0	0.9	0.1	0
3rd Sensor report: m3 (·)	0.58	0.07	0	0.35
4th Sensor report: m4 (·)	0.55	0.1	0	0.35
5th Sensor report: m5 (·)	0.6	0.1	0	0.3

**Table 2:** The negation of BPAs in the numerical example.

Negation of BPA	$\bar{m}(A)$	$\bar{m}(B)$	$\bar{m}(C)$	$\bar{m}(A,C)$
1st Sensor report: m1 (·)	0.295	0.355	0.35	0
2nd Sensor report: m2 (·)	0	0.1	0.9	0
3rd Sensor report: m3 (·)	0.21	0.465	0	0.325
4th Sensor report: m4 (·)	0.225	0.45	0	0.325
5th Sensor report: m5 (·)	0.2	0.45	0	0.35

**Table 3:** Fusion results with different methods in the numerical example.

Methods	m(A)	m(B)	m(C)	m(A,B)	m(A,C)	m(B,C)	m(A,B,C)
Ni et al.’s method [50]	0.6513	0.1648	0.1730	0.0016	0.0096	0.0016	0
Gan et al.’s method [51]	0.6881	0.1385	0.1572	0.0007	0.0074	0.0007	0
Deng et al.’s method [49]	0.9820	0.0039	0.0107	0	0.0034	0	0
Zhang et al.’s method [52]	0.9820	0.0033	0.0115	0	0.0032	0	0
Yuan et al.’s method [53]	0.9886	0.0002	0.0072	0	0.0039	0	0
The proposed method	0.9887	0.0007	0.0084	0	0.0037	0	0

**Table 4:** Fault diagnosis data modeled as BPAs in the application problem.

	Freq1				Freq2		Freq3			
BOE	{F2}	{F3}	{F1,F2}	{F1,F2,F3}	{F2}	{F1,F2,F3}	{F1}	{F2}	{F1,F2}	{F1,F2,F3}
m_s_1__(·)	0.8176	0.0003	0.1553	0.0268	0.6229	0.3771	0.3666	0.4563	0.1185	0.0586
m_s_2__(·)	0.5658	0.0009	0.0646	0.3687	0.7660	0.2341	0.2793	0.4151	0.2652	0.0404
m_s_3__(·)	0.2403	0.0004	0.0141	0.7452	0.8598	0.1402	0.2897	0.4331	0.2470	0.0302

**Table 5:** Uncertainty with the Deng entropy of BPAs in the application problem.

Ed(·)	Freq1	Freq2	Freq3
$E_{d}\left(m_{s_{1}}\right)$	1.1196	2.0146	2.0040
$E_{d}\left(m_{s_{2}}\right)$	2.3975	1.4422	2.2691
$E_{d}\left(m_{s_{3}}\right)$	3.0161	0.9784	2.1677

**Table 6:** Negation of BPAs for the original evidence in the application problem.

Negation of BPAs	Freq1				Freq2		Freq3			
	{F2}	{F3}	{F1,F2}	{F1,F2,F3}	{F2}	{F1,F2,F3}	{F1}	{F2}	{F1,F2}	{F1,F2,F3}
${\bar{m}}_{s_{1}}\left(\cdot \right)$	0.0608	0.3332	0.2816	0.3244	0.3771	0.6229	0.2111	0.1812	0.2938	0.3138
${\bar{m}}_{s_{2}}\left(\cdot \right)$	0.1447	0.3330	0.3118	0.2104	0.2341	0.7659	0.2402	0.1950	0.2449	0.3199
${\bar{m}}_{s_{3}}\left(\cdot \right)$	0.2532	0.3332	0.3286	0.0849	0.1402	0.8598	0.2367	0.1890	0.2511	0.3232

**Table 7:** Deng entropy of negation of the BPAs in the application problem.

E_d_(·)	Freq1	Freq2	Freq3
$E_{d}\left({\bar{m}}_{s_{1}}\right)$	3.1727	2.7047	3.3107
$E_{d}\left({\bar{m}}_{s_{2}}\right)$	3.0141	2.9352	3.2635
$E_{d}\left({\bar{m}}_{s_{3}}\right)$	2.6189	2.9985	3.2789

**Table 8:** Uncertain degree of the BPAs in the application problem.

E_du_(·)	Freq1	Freq2	Freq3
$E_{du}\left(m_{s_{1}}\right)$	4.2923	4.7193	5.3147
$E_{du}\left(m_{s_{2}}\right)$	5.4116	4.3774	5.5326
$E_{du}\left(m_{s_{3}}\right)$	5.6350	3.9769	5.4466

**Table 9:** Weight of body of evidence (BOE) in the application problem.

W_si_(·)	Freq1	Freq2	Freq3
$W_{s_{1}}\left(\cdot \right)$	0.2798	0.3610	0.3262
$W_{s_{2}}\left(\cdot \right)$	0.3528	0.3348	0.3396
$W_{s_{3}}\left(\cdot \right)$	0.3674	0.3042	0.3343

**Table 10:** Modified BPAs based on the proposed method for data fusion.

	Freq1				Freq2		Freq3			
	{F2}	{F3}	{F1,F2}	{F1,F2,F3}	{F2}	{F1,F2,F3}	{F1}	{F2}	{F1,F2}	{F1,F2,F3}
$m_{w}\left(\cdot \right)$	0.5167	0.0005	0.0714	0.4114	0.7429	0.2572	0.3113	0.4346	0.2113	0.0429

**Table 11:** Data fusion results for fault diagnosis in the application.

	Freq1				Freq2		Freq3			
	{F2}	{F3}	{F1,F2}	{F1,F2,F3}	{F2}	{F1,F2,F3}	{F1}	{F2}	{F1,F2}	{F1,F2,F3}
Results	0.8871	0.0002	0.0430	0.0697	0.9833	0.0170	0.3349	0.6323	0.0333	0.0002

**Table 12:** Data fusion results of different methods in the application under Freq1.

Method	Freq1						
	{F1}	{F2}	{F3}	{F1,F2}	{F1,F3}	{F2,F3}	{F1,F2,F3}
Ni et al.’s method [50]	0.1616	0.5051	0.1619	0.0587	0.0425	0.0425	0.0276
Gan et al.’s method [51]	0.1124	0.6408	0.1128	0.0591	0.0288	0.0288	0.0166
Jiang et al.’s method [54]	0	0.8861	0.0002	0.0582	0	0	0.0555
The proposed method	0	0.8871	0.0002	0.0430	0	0	0.0697

**Table 13:** Data fusion results of different methods in the application under Freq2.

Method	Freq2						
	{F1}	{F2}	{F3}	{F1,F2}	{F1,F3}	{F2,F3}	{F1,F2,F3}
Ni et al.’s method [50]	0.3938	0.3525	0.1697	0.0487	0.0162	0.0162	0.0030
Gan et al.’s method [51]	0.0666	0.7944	0.0666	0.0199	0.0199	0.0199	0.0121
Jiang et al.’s method [54]	0	0.9621	0	0	0	0	0.0371
The proposed method	0	0.9833	0	0	0	0	0.0170

**Table 14:** Data fusion results of different methods in the application under Freq3.

Method	Freq3						
	{F1}	{F2}	{F3}	{F1,F2}	{F1,F3}	{F2,F3}	{F1,F2,F3}
Ni et al.’s method [50]	0.1787	0.5278	0.1787	0.0348	0.0348	0.0348	0.0097
Gan et al.’s method [51]	0.4337	0.3679	0.1262	0.0498	0.0098	0.0098	0.0022
Jiang et al.’s method [54]	0.3384	0.5904	0	0.0651	0	0	0.0061
The proposed method	0.3349	0.6323	0	0.0333	0	0	0.0002

## Data Availability

All relevant data are within the paper.
